# Kynurenic acid as a biochemical factor underlying the association between Western-style diet and depression: A cross-sectional study

**DOI:** 10.3389/fnut.2022.945538

**Published:** 2022-10-10

**Authors:** Heather M. Francis, Richard J. Stevenson, Lorraine S. Y. Tan, Lauren Ehrenfeld, Sooin Byeon, Tuki Attuquayefio, Dolly Gupta, Chai K. Lim

**Affiliations:** ^1^Department of Psychology, Faculty of Medicine, Health and Human Sciences, Macquarie University, North Ryde, NSW, Australia; ^2^Department of Neurology, Royal North Shore Hospital, Sydney, NSW, Australia; ^3^Macquarie Medical School, Faculty of Medicine, Health and Human Sciences, Macquarie University, North Ryde, NSW, Australia

**Keywords:** Western-style diet (WSD), depression, kynurenic acid (KA), kynurenine pathway (KP), tryptophan metabolism, glutamatergic modulation, nutritional psychiatry

## Abstract

Consumption of a Western-style diet (WS-diet), high in saturated fat and added sugar, is associated with increased depression risk. However, the physiological mechanisms underlying the relationship requires elucidation. Diet can alter tryptophan metabolism along the kynurenine pathway (KP), potentially linking inflammation and depression. This study aimed to examine whether urinary inflammatory markers and KP metabolites differed according to WS-diet consumption and depression severity. Depression symptoms and habitual WS-diet consumption were assessed in 169 healthy adults aged 17–35 recruited from two experimental studies. Targeted metabolomics profiling of seven KP metabolites, ELISA-based assays of interleukin-6 (IL-6) and C-reactive protein (CRP) were performed using urine samples collected from the participants. Parametric tests were performed for group comparison and associations analysis. Multilevel mixed-effect modelling was applied to control for biases. Higher intake of WS-diet was associated with lower levels of neuroprotective kynurenic acid (KA; *R* = −0.17, *p* = 0.0236). There were no differences in IL-6 or CRP across diet groups (*p* > 0.05). Physical activity had negative associations with most KP metabolites. Mixed-effects regression analysis showed the glutamatergic inhibitor, KA, was the only biomarker to have a significant association with depression symptoms in a model adjusted for demographic and lifestyle variables: a unit increase in KA was associated with 0.21 unit decrease in Depression Anxiety and Stress Scale-21 depression score (*p* = 0.009). These findings suggest that urinary KA is associated with both habitual WS-diet intake, and levels of depression symptoms, independent of inflammation. Findings support the role of neuroprotection and glutamatergic modulation in depression. We propose that KA may act as endogenous glutamatergic inhibition in regulating depression severity in the absence of inflammation. Further comparison with blood-based markers will assist in validating the utility of non-invasive urine samples for measuring KP metabolites.

## Introduction

Depression, a common mental disorder, is a leading cause of disability and major contributor to overall burden of disease globally (1). Its onset is typically around mid- to late- adolescence and it is a major risk factor for suicide, which is the second leading cause of death in young adults. Depression is commonly managed with psychological interventions, which can be costly, and although various medications are available, many individuals experience unpleasant side effects or are treatment-resistant (2). There is a need for low-cost, low-risk interventions that can target lifelong modifiable risk factors for depression (3).

Diet is a modifiable risk factor for depression, offering a promising target for treatment and prevention. Consumption of Western-style diet (WS-diet), high in processed foods, saturated fat, and added sugars, is associated with increased risk of depression, whereas conversely, a healthy diet pattern is associated with reduced risk of depression (4). Randomised controlled trials have demonstrated that improvement in diet reduces clinical depression (5) and depression risk (6). In young adults, we showed that a brief dietary intervention for 3 weeks, which involved increasing intake of fruit, vegetables, and complex carbohydrates, while reducing the consumption of processed foods, improved depression symptoms in young adults (7).

**FIGURE 1:**
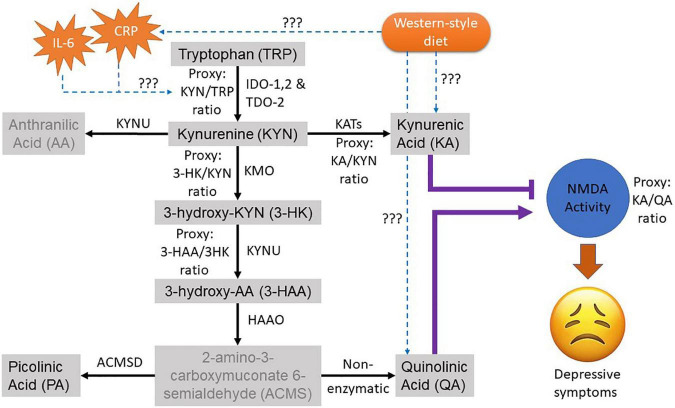
Overview of the kynurenine pathway (KP) of the tryptophan metabolism. The black arrow shows the natural route of the KP metabolism. Greyed metabolites indicate not measured in this study. The purple lines indicate the role of neuroactive KP metabolites on the glutamatergic NMDA activity where (T) indicates antagonistic and arrow indicate agonistic effect. The dotted blue arrow depicts the pathological interaction of interest with its effectors to be examined in this study. The ratio of the metabolites was used as proxies for their corresponding KP enzyme activities. CRP, C-Reactive protein; IL-6, interleukin-6; TRP, tryptophan; KYN, kynurenine; AA, anthranilic acid; KA, kynurenic acid; 3HK, 3-Hydroxykynurenine; 3HAA, 3-Hydroxyanthranilic acid; PA, Picolinic acid; QA, Quinolinic acid.

Although this accumulating evidence suggests dietary intervention may be an effective adjunct treatment for depression, there remains a need to understand the mechanisms underlying the relationship. There are several putative biochemical mechanisms that link diet quality and depression. A compelling theory is that WS-diet can alter the gut microbiota, influencing neurotransmitter metabolism, which can impact on brain function (8). This places tryptophan metabolism as an attractive target because of its multi-factorial role in diet, gut, and brain function. Tryptophan is an essential amino acid that is solely acquired through diet in vertebrates. The metabolism of tryptophan has been known for its important role in the regulation of many physiological functions, including immune activity, neurotransmission, mood, and behaviour (9). Catabolic routes of tryptophan *via* the serotonin-melatonin pathway and the kynurenine pathway (KP) produce neuroactive metabolites such as serotonin, kynurenic acid (KA), and quinolinic acid (QA) that alter serotonergic and glutamatergic neurotransmission, both of which are implicated in depression (Figure 1). Recent evidence suggests a strong interplay between tryptophan metabolism and the intestinal microbiota and host (10), and this relationship has been identified in people with depression and anxiety (11).

Another established theory, known as cytokine-induced sickness behaviour [see review (12)], involves inflammatory processes which can lead to increased production of pro-inflammatory mediators such as C-reactive protein (CRP) and interleukin (IL)−6. Again, the kynurenine-tryptophan pathway may be involved, as these pro-inflammatory mediators are strong inducers of the first enzyme in the KP, indoleamine 2,3 dioxygenase (IDO-1), which in turn activates the KP and alters tryptophan metabolism. Increased activation of IDO-1 can lead to decreased serotonin, thereby depleting the mood modulatory neurotransmitter [see serotonin hypothesis in review (13)], while increasing the *N*-methyl-D-aspartate (NMDA) agonist, QA, to exert an imbalance in (hypo)serotonergic and (hyper)glutamatergic neurotransmissions in depression. Moreover, WS-diet can increase pro-inflammatory cytokines, to drive this phenomenon (14). Taken together, it is possible that WS-diet can induce inflammatory processes that alter the host KP metabolism leading to the dysregulation of glutamatergic neurotransmission and depressive symptoms.

The above findings show it is important to gain greater understanding of the role of altered tryptophan metabolism down the KP in altering mood *via* dietary interventions (15, 16). Diet manipulations targeted at depression, including ketogenic and fasting diets, have been shown to modulate tryptophan-KP metabolism (17, 18). Further, individual diet components such as curcumin, resveratrol, black and green tea are associated with reduced depression symptoms and are also implicated in KP metabolism (19–22). High fat diets are associated with imbalanced intestinal flora, thereby affecting tryptophan metabolism (23), and supplementation with probiotics can modulate the KP activity (24, 25). Hence, tryptophan metabolism is a potential underlying physiological mechanism by which dietary intervention has the potential to improve mood.

We have previously conducted two randomised controlled trials that examined the relationship between diet quality and severity of depression in young adults, where baseline measures included fat and sugar intake, depression symptoms and a urine sample (amongst other measures). This offered the opportunity to measure KP metabolites, and immune markers in the urinary samples collected at baseline. We hypothesised that WS-diet intake is associated with proinflammatory mediators, CRP and IL-6, which in turn is associated with aberrant KP metabolism. Specifically, with regard to altered KP metabolism, we predicted decreased levels of tryptophan and/or increased kynurenine with a downstream consequence of enhanced glutamatergic activity defined by increased NMDA agonist, QA, and/or decreased NMDA antagonist, KA. We further hypothesised that these changes in the biochemical measures would be associated with the severity of depression symptoms. Using statistical modelling, we were able to examine the relationship between WS-diet intake and individual differences in inflammation and KP metabolism relating to the severity of depression in our sample of healthy young adults.

## Materials and methods

### Participants and study design

Participant data were utilised from the baseline testing session of two separate studies where we obtained diet quality information using the Dietary Fat and Sugar Screener (DFS) and level of depression symptoms using the Depression, Anxiety and Stress Scale-21 (DASS-21). There were differences in the selection criteria for each study (outlined below), which reflected the overall aims. Study 1 (*n* = 100) aimed to recruit individuals who habitually consume a WS-diet and with elevated depression symptoms (7), whereas Study 2 (*n* = 73) aimed to recruit individuals with healthy diet and non-elevated levels of depression symptoms (26). By combining the baseline testing data from the two studies, we were able to produce an overall sample with variation in dietary fat and sugar intake and DASS-21-depression (DASS-21-D) symptoms. Participants from both studies were either recruited from an undergraduate psychology course, and participated for course credit, or *via* advertisement on campus and surrounds and participated for cash reimbursement.

#### Selection criteria

Study 1 inclusion criteria: aged 17–35 years, score of ≥ 7 on the DASS-21-D, which corresponds with moderate or higher depression symptoms (27), and a score of > 57 on the Dietary Fat and Sugar Screener (DFS), with scores > 57 representing higher intake of WS-diet that does not comply with the Australian Guide to Healthy Eating (28). If receiving antidepressant medication or psychological therapy, participants were required to be on the same treatment for at least 2 weeks before study participation. Study 2 inclusion criteria: aged 17–35 years, body mass index (BMI) between 17 and 26, DFS score < 57, and an overall depression score below 25 (27).

Exclusion criteria for both studies were similar but with slight differences. Study 1 exclusion criteria: pregnancy, currently dieting, history of eating disorders or metabolic disease(s), history of psychological illness other than depression or anxiety, medical condition that could be adversely affected by diet change, poor proficiency in English, recent illicit drug use, or sickness in the past week. Study 2 exclusion criteria: pregnancy, current/past metabolic, neurological or psychiatric illnesses, food allergies, vegan/vegetarian, non-pork eater, currently dieting, recent significant diet change, prescription medication use (other than the contraceptive pill and asthma medication), poor proficiency in English, illicit drug use, and current ill-health.

#### Ethics approval

Both studies were conducted according to the guidelines laid down by the Declaration of Helsinki, with written informed consent obtained for each participant. The studies protocols were approved by the Macquarie University Human Research Ethics Committee (5201822302603 and 5201600641).

### Baseline measurement

Biographical and health data were collected during the baseline sessions of each study. Body measurements (height and weight) were taken to calculate BMI and a urine sample was provided. Current levels of physical activity were calculated using the International Physical Activity Questionnaire (29) and sleep quality using two items from the Pittsburgh Insomnia Rating Scale (30). Intake of saturated fats and added sugar was measured using the Dietary Fat and Sugar Screener (DFS) (28). In the DFS, participants were asked to rate how often they had consumed 26 food and drink items over the past year on a five-point category scale ranging from 1 = “Less than one time a month” to 5 = “Five or more times a week.” Depression symptoms were assessed using the DASS-21-D, a self-report measure rated on a 4-point Likert scale regarding mood over the past week (27).

### Biochemical measurement

#### Reagents

Analytical grade reagents and standards were purchased from Sigma-Aldrich (St Louis, MO, USA), unless otherwise stated. Deuterated internal standards were purchased from Medical Isotopes, Inc (Pelham, NH, USA).

#### Creatinine measurement

Mid-stream urine samples were collected for the determination of urinary creatinine concentrations using a Creatinine (urinary) Colorimetric Assay kit (Cayman Chemical, Ann Arbor, MI, USA) according to the manufacturer’s instructions. The creatinine concentration was used as an index of normalisation to control for between-participant variability in excretion rate.

#### Immune marker profiling

Blinded urine samples were subjected to immunoassays to quantify for Interleukin-6 (IL-6) and C-reactive protein (CRP) using the Human IL-6 (ab178013, Abcam, Boston, MA, USA) and CRP (ab260058, Abcam, Boston, MA, USA) ELISA kits following the manufacturer’s instructions.

#### Kynurenine pathway metabolites profiling

Before analysis, blinded urine samples were deproteinized with respective mobile phase of the KP assays at a dilution factor of 1:10. Samples were incubated for 5 min, vortexed and then centrifuged (4°C) for 10 min at 12,000 rpm. Supernatant was then extracted and filtered with syringe filters (0.22 μm) ready for injection into analysers.

Concurrent quantification of TRP, KYN, 3-HK, and 3HAA using an Agilent 1290 ultra-high performance liquid chromatography system while PA and QA were analysed using an Agilent 7890 gas chromatograph (GC) coupled with an Agilent 5975 mass spectrometer according to the method described in Lim et al. (31). KA quantification was conducted using an Agilent 1260 system as previously described in Jacobs et al. (32). Mixed standards of all metabolites were used for a six-point calibration curve to interpolate the quantity of the sample readout. The inter- and intra-assay coefficient of variation was within the acceptable range of 3–7% for the LC system and 5–10% for the GC system. Participant’s urinary KP metabolites measurements were normalised with their corresponding creatinine levels. Five ratios were calculated based on the metabolite levels, namely, the kynurenine/tryptophan ratio, which depicts combined IDO and tryptophan dioxygenase activity, kynurenic acid/kynurenine ratio depicts kynurenine aminotransferase activity, 3-hydroxykynurenine/kynurenine ratio depicts kynurenine monooxygenase activity, 3-hydroxyanthranilic acid/3-hydroxykynurenine ratio depicts kynureninase activity, and kynurenic acid/quinolinic acid depicts KP-induced glutamatergic activity.

### Statistical analysis

Prior to statistical analysis, all continuous variables were checked for normality. Data were log-transformed and scaled, where appropriate, to achieve a normal distribution for parametric analysis. The nature of the missing values was confirmed and treated as missing completely at random. The outliers were examined based on DFBETA using bubble plots to look for overly influential observations.

Univariate analysis (unadjusted for covariates) was used for the descriptive statistics. For group comparisons between the three DFS groups, one-way ANOVA with Sidak multiple-comparisons *post-hoc* tests was used. Data are expressed as mean ± SD unless otherwise specified and *p* < 0.05 (two tailed) was considered statistically significant. Biochemical measures that showed differential expression were considered for further analysis. Apart from exploring associations among biochemical measures, potential confounders were identified using pairwise Pearson’s correlations between biochemical measures and demographic factors. Any significant correlations were subsequently adjusted in the multiple regression models described below.

The univariate regression model was applied to tease out the association between each selected biochemical measure with the depression scores as outcomes, followed by a covariate regression analysis adjusting for potential confounding effects from the demographic factors. Models were checked for multicollinearity and variance inflation factors were < 3.

Two main models were created to examine the impact of both lifestyle factors and biochemical markers on the severity of depressive symptoms using multiple linear regression. The difference in the two models was the presence and absence of immune marker(s). The models comprised the DASS-21 depression score as dependent variable and three groups of predictors: (1) demographic factors, (2) lifestyle factors, and (3) urinary biochemical measures, as defined below:

Model 1 (only KP markers):


Depression⁢score=Demographic⁢factors+Lifestyle⁢factors+KP⁢markers

Model 2 (KP and immune markers):


Depression⁢score=Demographic⁢factors+Lifestylefactors +Immune markers+KP⁢markers

The beta coefficients infer the unit change in (DASS-21) depression scores after adjusting for all other covariates in the model. The *R*^2^ value depicts the goodness-of-fit of the regression model together with corresponding p values. Biochemical measures with *p* < 0.05 were considered important predictors of the outcome. To further control for the random effect from the two separate studies, we further modelled the outcome and selected predictors in Model 2 using mixed-effects multilevel linear regression. The intraclass correlation of the model was used to check for (in)dependency between the two RCTs.

All statistical analyses were performed in StataIC 16 (StataCorp LLC, CA, USA) and graphical illustrations were prepared with GraphPad Prism 9.0 (GraphPad Software, Inc, La Jolla, CA, USA).

## Results

### Group characteristics

Of the 173 participants recruited, four participants were removed due to lack of or inadeqaute sample volume for analysis and were treated as missing completely at random. A flow diagram outlining other missing values was presented in Supplementary Figure 1. Hence, a total of 169 samples were analysed that were classified into low (DFS score < 54), mid (DFS score >54 and < 66), and high (DFS score >66) DFS groups as outlined in Table 1. Comparison of demographic characteristics between the three DFS groups showed no differences in age or the frequencies of female-to-male ratio (χ^2^ = 2.70, *p* = 0.758) although there was a slight tendency for a higher proportion (52–67%) of female participants across the groups (Table 1). Other lifestyle-associated factors such as BMI and physical activity level were well-matched between the three DFS groups, showing no differences (*p* > 0.05) except for DFS itself [*F*_(2,__166)_ = 310.11, *p* < 0.0001]. There was no difference in the urinary creatinine level, suggesting that the diet did not affect the urinary excretion rate. The DASS-21-D score was significantly different between the DFS groups [*F*_(2,__166)_ = 11.99, *p* < 0.0001], with highest level of depression being observed in the high DFS group (Table 1).

**TABLE 1:** Demographic characteristics of cohort participants based on DFS score.

	Low DFS (*n* = 68)	Mid DFS (*n* = 55)	High DFS (*n* = 46)	F-statistic or χ^2^ value	*P*-value
Age (years, mean ± SD)	20.97 ± 3.11	22.05 ± 4.48	20.80 ± 3.79	1.71^A^	0.185
Sex (F/M, % of F to total)	38/30 (55.88)	37/18 (67.27)	24/22 (52.17)	2.695^B^	0.260
BMI (mean ± SD)	22.14 ± 2.63	21.78 ± 2.82	22.19 ± 2.89	0.36^A^	0.701
Physical activity (mean ± SD)^¥^	12.09 ± 1.92	12.04 ± 1.65	11.84 ± 1.12	0.32^A^	0.725
DFS (mean ± SD)	45.44 ± 5.20	59.85 ± 3.22^$^	76.28 ± 11.58^$^	310.11^A^	** < 0.0001**
Creatinine, mmol/L (mean ± SD)	15.25 ± 10.08	13.14 ± 7.08	10.89 ± 6.42	1.91^A^	0.151
DASS-21-D (mean ± SD)	2.87 ± 3.50	5.29 ± 4.71^∧^	6.91 ± 5.65^$^	11.99^A^	** < 0.0001**

DFS, dietary fat and free sugar screener score; DASS-21-D, Depression Anxiety Stress Scales 21 Depression score; BMI, Body Mass Index; F, Female; M, Male. ^A^Denotes One-way ANOVA and ^B^denotes Chi-square analysis. Significant p-value (< 0.05) are denoted in bold. ^∧^Denotes p = 0.001 and ^$^denotes p < 0.0001 using Sidak post-hoc analysis in comparison to low DFS group. ^¥^Denotes the mean of variable presented in log_2_-scale. All post-hoc between groups comparisons have p < 0.0001 in 1-way ANOVA for DFS.

### Potential confounding effects between demographic factors and urinary biomarkers

We examined the immune markers and KP metabolites for any potential confounding effects with the cohort’s demographic factors. Summarised in Table 2, we found that increased age was associated with an elevation in several KP metabolites and enzyme activities including KA, 3HAA, PA, KAT, KMO, KYNU, and KA/QA ratio. Our cohort showed a moderately strong effect size of sex differences in urinary QA level (Cohen’s *D* = 0.51, *p* = 0.0013) and KMO activity (*D* = 0.49, *p* = 0.0019), being higher in males, while KA/QA ratio (*D* = −0.36, *p* = 0.0235) was higher in females.

**TABLE 2:** Univariate analysis of associations between demographic, lifestyle risk factors, and urinary biomarkers.

	Age	Sex^A^	BMI	Physical activity	DFS
**Immune markers**					
C-Reactive Protein (CRP)	−0.02	0.23	0.03	0.15*	0.07
Interleukin (IL)-6	0.03	0.29*	−0.01	0.08	0.00
**KP metabolites**					
Tryptophan (TRP)	−0.05	0.00	0.08	−0.17*	0.08
Kynurenine (KYN)	−0.12	−0.16	−0.05	−0.16*	−0.01
Kynurenic acid (KA)	**0.27^**	0.01	0.08	−0.15*	−0.17*
3-Hydroxykynurenine (3HK)	0.02	0.22	0.06	−0.15*	0.13
3-Hydroxyanthranilic acid (3HAA)	**0.23^#^**	0.09	−0.01	−0.16*	**0.16** *
Picolinic acid (PA)	**0.24^#^**	−0.22	0.06	−0.10	0.11
Quinolinic acid (QA)	−0.01	**0.51** *	−0.02	−**0.24^#^**	0.08
**KP activity/ratio**					
IDO/TDO activity	−0.06	−0.15	−0.12	0.02	0.08
KAT activity	**0.29** ^$^	0.13	0.10	0.01	−0.13
KMO activity	**0.16** *	**0.49** *	0.13	−0.03	**0.19** *
KYNU activity	**0.19** *	−0.11	−0.07	−0.01	0.03
KA/QA ratio	**0.25^**	−0.36*	0.09	0.03	−**0.22^#^**

IDO, Indoleamine 2,3-dioxygenase; TDO, Tryptophan dioxygenase; KYAT, Kynurenine aminotransferase; KMO, Kynurenine 3-monooxygenase; KYNU, Kynureninase; IDO/TDO activity is defined by KYN/TRP ratio; KAT activity is defined by KA/KYN ratio; KMO activity is defined by 3HK/KYN; KYNU activity is defined by 3HAA/3HK ratio. All reported values were derived from Pearson’s correlation coefficient unless otherwise specified. ^A^Denotes Cohen’s D effect size (female to male). Significant correlations/effect sizes are denoted in bold *p < 0.05, #p < 0.01, ^p < 0.001, $p < 0.0001. *(unbold) indicate p-value close to 0.05.

For lifestyle factors, there was no association between BMI and any biochemical measure. The correlational analysis indicated that physical activity level was negatively correlated with all of the KP metabolites, except PA (Table 2). Among the KP metabolites, DFS scores were negatively associated with KA (*R* = −0.17, *p* = 0.0236) and positively associated with 3HAA (*R* = 0.16, *p* = 0.0386). Higher KMO activity (*R* = 0.19, *p* = 0.016), but lower KA/QA ratio (*R* = −0.22, *p* = 0.0037) were correlated with increasing DFS score. Taken together, our univariate correlational analysis indicated that subsequent regression modelling using the urinary KP biomarkers should be adjusted for confounding effects with age, sex, physical activities, and DFS score.

### Correlation analyses of immune and kynurenine pathway markers

We found that urinary CRP was correlated with IL-6 (*R* = 0.20, *p* = 0.008), although the effect size was small (Table 3). This is not surprising considering the relatively healthy population of our cohort with the expectation of more regulated inflammatory responses. The only KP metabolites that correlated positively with CRP levels were 3HK (*R* = 0.20, *p* = 0.011) and 3HAA (*R* = 0.28, *p* = 0.0002), showing a small effect size with statistical significance. We found that IL-6 correlated positively with the key KP metabolite involved in depression, KA, although the effect size was small (*R* = 0.20, *p* = 0.0079). Neither TRP nor KYN correlated with IL-6 despite the presence of depressive symptomology (>7 DASS-21-D scores) in ∼60% of participants.

**TABLE 3:** (Inter-)Relationship between immune markers and KP metabolites.

	IL-6	TRP	KYN	KA	3HK	3HAA	PA	QA
CRP	**0.20^#^**	0.08	0.06	−0.03	**0.20** *	**0.28^**	0.07	0.10
IL-6								
TRP	0.07							
KYN	0.01	**0.45^$^**						
KA	**0.20^#^**	−0.02	0.14					
3HK	−0.03	**0.49** ^$^	**0.70** ^$^	0.09				
3HAA	0.04	0.08	**0.30** ^$^	**0.21** *	**0.40** ^$^			
PA	0.03	0.14	**0.18** *	**0.24^#^**	0.15	**0.37** ^$^		
QA	0.09	0.13	**0.25**^	**0.29**^	**0.31** ^$^	**0.20** *	**0.29**^	

Significant correlations are denoted in bold. *p < 0.05, #p < 0.01, ^p < 0.001, $p < 0.0001. CRP, C-Reactive protein; IL-6, interleukin-6; TRP, tryptophan; KYN, kynurenine; KA, kynurenic acid; 3HK, 3-Hydroxykynurenine; 3HAA, 3-Hydroxyanthranilic acid; PA, Picolinic acid; QA, Quinolinic acid.

As there is limited knowledge about KP homeostasis in adolescent/young adult cohorts, we used correlational analyses to study baseline KP metabolism in this age group. Our analysis indicated that in general, TRP positively correlates with its downstream KP metabolites suggesting a normal metabolic flux of more substrates, leading to more catabolites. We saw that the key branching metabolite of the KP, KYN, was highly associated (*R* = 0.70, *p* < 0.0001) with catabolites of the 3HK branch towards QA and not KA production. Other KP intermediates such as 3HK and PA, also showed moderate effects with QA. There was a moderate correlation between QA and KA, suggesting that increments in either one are likely to result in increases in its NMDA counterpart. Additionally, higher 3HAA, a precursor to PA and QA, was more likely to be associated with PA, rather than QA production.

### Differential group expression of urinary kynurenine pathway metabolites

**FIGURE 2:**
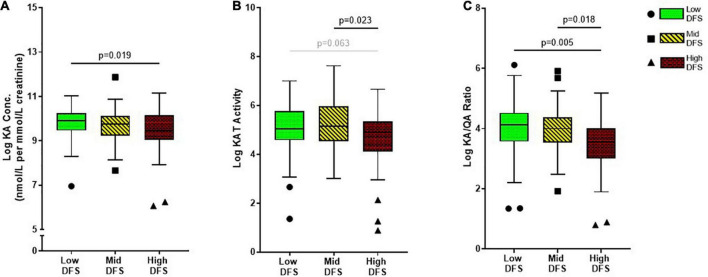
Boxplots showing the difference in **(A)** Kynurenic acid (KA), **(B)** Kynurenine aminotransferase (KAT) activity, and **(C)** KA/QA ratio across the low, mid, and high DFS groups. The low DFS participants are represented by green box-and-whisker and circle symbols, the mid DFS participants are represented by yellow box-and-whisker and square symbols, and the high DFS participants are represented by red box-and-whisker and triangle symbols. The symbols indicated participants that had > 2 standard deviations within the group. The median and interquartile range are displayed for each group.

There were group differences [*F*_(2, 166)_ = 3.86, *p* = 0.023] in urinary KA levels, which were the highest in the Low DFS group compared to other groups, with significant differences in the High DFS group, as shown in Figure 2A. Next, we examined the overall kynurenine aminotransferase (KAT) activity defined by the ratio of KA to kynurenine. As expected, there were group differences [*F*_(2, 166)_ = 4.10, *p* = 0.018] in KAT activity as well. The high DFS group had the lowest KAT activity, and was significantly lower than the Mid DFS group and the Low DFS group, although the latter did not reach statistical significance (Figure 2B).

As QA and KA are the two key metabolites of the KP known to be associated with depression, with depressed individuals showing higher neurotoxic QA levels and lower neuroprotective KA levels. Considering this relationship with depression, we were interested in whether this ratio would differ across DFS group, representing a potential mechanism underlying the relationship between WS-diet intake and depression. We found significant differences in KA/QA ratio between the DFS groups [*F*_(2, 166)_ = 5.70, *p* = 0.004]. Although there was no difference between Low DFS and Mid DFS groups, the High DFS group had a significantly lower KA/QA ratio compared to the Low DFS group and Mid DFS group (Figure 2C). None of the urinary immune markers or other KP markers showed any group differences, as outlined in Supplementary Table 1.

### Assessing depression with urinary biomarkers and demographic factors

Our results indicate that IL-6, but not CRP, was associated with the severity of depressive symptoms. A unit increase in urinary IL-6 was associated with a decrease of 0.041 units of depression score (*p* = 0.013), after adjusting for demographic factors (Table 4).

**TABLE 4:** Associations between depression and biomarkers measures by univariate and covariate analyses.

	Univariate analysis			Covariate analysis*		
	β (95% CI)	*R* ^2^	*P*-value	Adjusted β (95% CI)	Adjusted *R*^2^	*P*-value
**Immune marker**						
IL-6	−0.04 (−0.07 to −0.01)	0.04	**0.013**	−0.04 (−0.07 to −0.01)	0.15	**0.013**
**KP markers**						
KA	−0.30 (−0.46 to −0.14)	0.08	**<0.001**	−0.27 (−0.43 to −0.10)	0.17	**0.002**
KAT activity	−0.16 (−0.28 to −0.03)	0.04	**0.014**	−0.10 (−0.23 to 0.03)	0.13	0.123
KMO activity	0.18 (0.004–0.36)	0.02	**0.045**	0.14 (−0.04 to 0.33)	0.13	0.121
KA/QA ratio	−0.29 (−0.44 to −0.14)	0.08	**<0.001**	−0.22 (−0.37 to −0.06)	0.16	**0.006**

IL-6, Interleukin-6 (fg/mL per mmol/L creatinine); KA, Kynurenic acid (nmol/L per mmol/L creatinine); KAT, Kynurenine aminotransferase; KMO, Kynurenine 3-monooxygenase; KA/QA, Kynurenic acid/Quinolinic acid ratio; KAT activity is defined by KA/KYN ratio; KMO activity is defined by 3HK/KYN; *Multiple linear regression modelling was performed to adjust for demographic factors including age, sex, DFS, and physical activity. All variables were log_2_-transformed prior to regression analysis. The β denotes the beta coefficient of the regression model and R^2^ refers to the coefficient of determinant indicating the goodness-of-fit of the regression analysis. Significant p-value (< 0.05) are denoted in bold. Only biomarkers that showed a significant relationship with depression score were presented in this table. For full analysis of the other biomarkers, refer to Supplementary Table 2.

Among all KP markers, KA, KAT activity and KA/QA ratio had significant negative correlations with the severity of depression (Supplementary Table 2). However, only KA and KA/QA ratio remained to be significant biomarkers of depression after adjusting for demographic factors: one unit increase in KA levels and KA/QA ratio were associated with a decrease of 0.269 units (*p* = 0.001) and 0.220 units (*p* = 0.005) in depression score, as outlined in Table 4. Conversely, KMO activity showed a positive correlation with the severity of depression, but did not reach statistical significance when adjusted for confounding effects.

### Relationship between depressive symptoms with diet, exercise, and kynurenic acid

We were interested to know whether lifestyle (i.e., diet and exercise) and intrinsic (i.e., host’s biochemical measures) factors were related to severity of depressive symptoms defined by DASS-21-D scores. After a thorough interrogation of the dataset, we selected the important predictors in building our model using multivariate regression analysis. Two models were established, where in Model 1, only the KP metabolites but not immune mediator were used as the intrinsic biomarker and in Model 2, both immune and KP biomarkers were included. As the data were taken from the baseline session of two separate RCTs, there may be random effects (i.e., study factors) that were not taken into account in Models 1 and 2. Hence, a multilevel mixed-effect regression model was applied to adjust for random effects. Our analysis showed that there was no significant differences between the β-coefficients of the predictors when comparing Model 2 and 3 (Supplementary Table 3).

After adjusting for demographic factors (i.e., age, sex, and BMI) and random effects from the two separate RCTs, our final model (i.e., Model 3) showed that increasing physical activity was associated with reduced severity of depressive symptoms (Table 5). Every additional hour of physical activity is associated with a lower depressive symptom score of 5.4 units. Not surprisingly, both Mid and High DFS groups were associated with higher depression scores compared to the low DFS group, as shown in Table 1. In addition, every 1 unit increase in DFS score was associated with 0.69 units increase in depression score.

**TABLE 5:** Depression severity correlates with diet, physical activity, and the KP.

	Regression model β coefficient (95% CI) *n* = 166	*P*-value
**Demographic factors**		
Age (Years)	−0.25 (−0.80 to 0.30)	0.377
Sex	0.01 (−0.25 to 0.27)	0.929
BMI	−0.02 (−0.07 to 0.02)	0.341
**Lifestyle factors**		
Physical activity	**−0.09 (−0.16 to −0.01)**	**0.030**
DFS (as score)	**0.69 (0.32 to 1.05)**	**<0.001**
Low Group¤ (score < 54)	Reference	
Mid Group¤ (score 54–65)	**0.51 (0.22–0.80)**	**0.001**
High Group¤ (score ≥ 66)	**0.56 (0.26–0.87)**	**<0.001**
**Immune marker**		
IL-6, fg/mL per mmol/L Cr	−0.03 (−0.06 to 0.00)	0.077
**KP marker**		
KA, nmol/L per mmol/L Cr	−**0.21 (**−**0.37 to**−**0.05)**	**0.009**

The selected model was analysed using Mixed-effect multilevel regression to control for random effects from different studies. BMI, Body Mass Index; DFS, dietary fat and free sugar screener score; IL-6, interleukin-6; KA, kynurenic acid; Cr, creatinine. All variables except BMI were log_2_-transformed prior to analysis. ¤ denotes variable was chosen to be analysed primarily as a categorical variable rather than continuous variable. Only variables with p-values < 0.05 were considered important in predicting the severity of the depressive symptoms. Significant p-value (< 0.05) are denoted in bold.

When accounting for the covariates, the immune marker IL-6 no longer became an important predictor of the model (*p* > 0.05). More importantly, only one biomarker, KA, showed a significant association with the severity of depressive symptoms, regardless of the presence or absence of IL-6. Our model indicates that with every decrease in 1 μM of the NMDA antagonist, KA level in urine, depression scores increased by 0.21. Our model could explain approximately 22% of the variance in relation to how diet, physical activity, and the KP impact on depression severity in an otherwise healthy young adult cohort.

## Discussion

Depression is a common mental health disorder, with approximately 1 in 5 people experiencing at least one episode in their lifetime. An association between diet and depression has been demonstrated across numerous studies, and several RCTs have implied that eating a more healthful diet is a useful adjunct treatment for depression. However, the physiological mechanisms by which diet can impact on depression symptoms have yet to be established. This was the first study to examine and provide evidence to support the biochemical relationship between the urinary KP metabolites with WS-diet and depressive symptoms in otherwise healthy young adults. Overall, we showed that (a) WS-diet was associated with alterations to the KP profile, (b) the KP profile was associated with severity of depression symptoms, and (c) after adjusting for confounding effects, including diet, KA and KA/QA ratio remained strong predictors of level of depression symptoms. These findings are discussed in more depth below.

We found that higher saturated fat and sugar intake was associated with lower levels of neuroprotective KA. To understand the metabolic homeostasis associated with differing levels of saturated fat and sugar intake, we further examined the conversion of KYN to KA, operationalised as KAT activity, across the DFS groups. We found higher KAT activity in those who have healthier diet reflected by lower DFS score, whereas KYN levels remained consistent across groups (Figure 2B). This suggests that reduced consumption of WS-diet is linked to increased KAT activity, which may confer neuroprotection against depression. As hypothesised, higher WS-diet intake was also associated with a lower KA/QA ratio. Interrogating this relationship, we found that although levels of KA were correlated with that of QA, DFS score was only associated with KA (negatively) but not QA production, implying that WS-diet selectively alters the inhibitory glutamatergic activity by limiting the NMDA antagonist, KA. Hence, our study showed that, in otherwise healthy young adults, WS-diet was associated with lower levels of KA, KAT activity, and KA/QA ratio.

The glutamatergic modulators (i.e., KA and KA/QA), which were negatively correlated with WS-diet intake, were also negatively correlated with depressive symptoms. The role of QA and KA in depression, in the context of glutamatergic modulation, is well-known, though it is debatable whether QA or KA plays a more important role. A recent meta-analysis showed that reduced KA, but no change in QA was associated with MDD, which is consistent with our findings (33). It is possible that the diet plays a direct role in regulating KA levels, while QA levels are linked to inflammation in the pathology of depression; explaining why we did not observe a significant increase in QA levels. It is also possible that intestinal microbes can directly affect KA levels in the gut and influence the host’s peripheral KA levels since microbes have aspartate aminotransferase, in addition to KAT (limited to vertebrates), which is an alternative to KA production *via* the transamination reaction. Hence, the diversity and capability of the host’s gut microbiota to produce KA can contribute to differences in hosts’ KA levels. However, our understanding of the crosstalk between luminal and peripheral KA levels is limited and warrants further investigation. Hence, our findings were consistent with the current literature supporting the dysregulation of the glutamatergic system and challenges the long-held belief that serotonin as the key neurotransmission system involved in depression.

Though the mechanisms of action underlying relationship between diet and depression have yet to be fully understood, tryptophan metabolism down the KP pathway has been of increasing interest. Tryptophan depletion by oral intervention in healthy male participants was shown to lead to depression (23). This was attributed to attenuation of the mood regulating neurotransmitter, serotonin and interpreted as support for the ongoing theory of tryptophan-serotonin depletion in depression (13). However, further studies showed the involvement of the glutamatergic-acting KP metabolites (as opposed to the other arm; the serotonin pathway) in people with severe depression (34). Our findings contribute to an emerging literature demonstrating that the pathophysiology of depression extends beyond serotonergic modulation. Our study further suggests that in otherwise healthy young adults, KA may be an endogenous source of glutamatergic inhibition, acting to reduce depression. This notion is supported by recent studies showing glutamatergic inhibition by ketamine is an effective antidepressant (35–37).

One of the putative mechanisms for how diet can impact depression, which has garnered a lot of interest, is alterations to the gut-brain axis. The involvement of tryptophan-KP is congruent with this theory. WS-diet can directly alter the host’s tryptophan (and therefore KP) metabolism through the interaction between diet composition and gut microbiota. Short-chain fatty acids (SCFAs), especially butyrate, are functional by-products from bacterial carbohydrate metabolism in the gut known to modulate the KP. Specifically, butyrate can inhibit IDO-1 activity in the gut environment to increase the bioavailability of luminal tryptophan in the host (38). This is consistent with a study by Gao et al. (39) which demonstrated increased luminal availability of carbohydrates by cecal starch infusion leads to suppression of tryptophan catabolism in the microbial environment, thereby resulting in greater bioavailability of tryptophan in the large intestine and subsequently higher level in hosts’ serum (39). Similarly, another preclinical study showed that high-fat diet can attenuate microbial tryptophan degradation in the cecum of mice (40). This is in agreement with our results showing that participants with the highest levels of fat and sugar intake had the highest level of urinary tryptophan compared to Low and Mid DFS groups, although the group comparison was not statistically significant.

Given that WS-diets are known to induce systemic low-grade inflammation, we explored pro-inflammatory mediators (IL-6 and CRP) that are known to activate the KP *via* IDO-1 to establish the correlations between diet, inflammation and KP changes. There were no group differences in these pro-inflammatory cytokines across diet groups (Supplementary Table 1) and demographic factors did not correlate significantly with these proinflammatory cytokines in our cohort. As expected, IL-6 was a predictor of depression symptoms in our univariate regression model, although, to our surprise, urinary IL-6 had an inverse correlation with level of depression symptoms. This is inconsistent with the widely accepted role of IL-6 and inflammation in the pathogenesis of MDD [see review (41)]. However, this research is based on serum levels of IL-6, whereas in the current study we only examined urine levels. While we expected that peripheral markers would correlate positively with urine markers, most of the studies showing a positive correlation between serum and urine IL-6 levels have been conducted in patients with renal dysfunction. It may be the case that the relationship is different in otherwise healthy young adults. A possible explanation is that the immune markers in urine represent what is excreted from the host, as opposed to the blood-based profile, which directly reflects the host’s systemic condition. Hence, the concentration of urinary markers would be inversely proportional to periphery markers. Our recent study comparing serum and matching urine immune markers between healthy controls and people with multiple sclerosis (MS) showed higher levels of immune markers (IP-10, IL-1ra, TNF-alpha, and RANTES) in the serum of people with MS compared to healthy controls, whereas, in urine, the result was the opposite to the matching serum (42). In our study, it is possible that healthy individuals who are less susceptible to depression may have greater efficiency in excreting immune metabolites. The lack of serum blood samples is thus a limitation of the current study, and future studies collecting both serum and urine samples may shed further light on this proposition. Furthermore, we are aware of only a few studies analysing urinary KP metabolites as a diagnostic biomarker in conditions such as attention-deficit hyperactivity disorder (43), cardiovascular events (44), heart failure (45), and breast cancer (46). That we could observe significant differences in KP metabolites according to both depression and WS-diet reveals further promise for urine based biomarkers as a less invasive and more affordable method of examining physiological mechanisms. For example, as a prognostic marker in predicting risk of a major depressive episode or suicidal ideation in younger people with depressive symptoms. However, this too requires replication in another cohort as well as validation against blood samples.

Though both inflammation and KP metabolism are implicated in the pathophysiology of depression, it is rather controversial as to whether inflammation is needed to drive the dysregulation of the KP activity (*via* IDO-1). For example, Öztürk et al. (47) showed that the KYN/TRP ratio and QA were significantly higher in MDD, but did not find any group differences in IL-6 and CRP levels compared to healthy controls (47). Contrary to this, Erhardt et al. (48) found higher IL-6 and QA in CSF of people with MDD compared to healthy controls (48). As such, KP related changes in depression may be independent of inflammation, and our findings support this notion based on the observations of: (a) an inverse relationship between IL-6 and depressive symptoms, (b) no inverse correlation between tryptophan and its downstream metabolites or differential in KYN/TRP ratio, an indicator of host IDO/TDO activity, (c) limited associations between the pro-inflammatory and KP markers, and (d) KA was the only biomarker to remain a significant predictor of depression severity in a model accounting for covariates including IL-6. Although the lack of proinflammatory status may imply that competition for tryptophan as substrate between the kynurenine and serotonin pathway may not be affected, however, future study should consider exploring serotonin and downstream metabolites such as 5HIAA in urine to delineate the metabolic flux of tryptophan metabolism in dietary intervention in the context of depression.

A number of expected findings emerged, which make us more confident in our data. Age was associated with an increase in several KP metabolites, which is in keeping with previous findings (49, 50). Age may be a confounder in prevalence and pathogenesis of depression, thereby limiting our findings to the young adult population. We also found some sex differences, such that QA levels and KMO activity were higher in males, whereas KA/QA ratio was higher in females. Similarly, higher urinary levels of KYN, 3-HAA, and TRP in males compared to females, in young adults aged 20–24 (51). Our results additionally demonstrated that increased physical activity was associated with decreased severity of depressive symptoms, as well as decreased levels of KP metabolites. Consistent with this, a recent study showed reduced urinary KP metabolites in those who exercise compared to those who do not exercise (46). Future clinical studies that examine KP metabolism should consider physical activity as a potential confounder.

In conclusion, this is the first study to examine the role of KP metabolism in the relationship between WS-diet and depression symptoms in an otherwise healthy young adult cohort. Our findings showed that higher WS-diet intake was related to reduced production of the neuroprotective KP metabolite, KA, as well as a reduced KA/QA ratio. These glutamatergic modulators, were also shown to be reduced in those with higher levels of depression symptoms, with further analysis showing KA was the only biomarker to have a significant association with depression symptoms in a model controlling for demographic and lifestyle variables. These findings appeared to be independent of inflammation, however, further comparisons between urine and blood based immune biomarkers are required. Animal studies may be helpful to investigate direction of causality in the relationship between depression symptoms and KP metabolism.

## Data availability statement

The raw data supporting the conclusions of this article will be made available by the authors, without undue reservation.

## Ethics statement

The studies involving human participants were reviewed and approved by Macquarie University Human Research Ethics Committee (Ethics #5201822302603 and 5201600641). The patients/participants provided their written informed consent to participate in this study.

## Author contributions

HF, RS, and CL contributed to the conception, design, supervision, and funding acquisition of the study. LE, TA, and DG were involved in the investigation and collection of the clinical data. LT and SB were involved in performing and collecting the biochemical data. CL and SB performed the statistical analysis. CL and HF wrote the first draft of the manuscript. LT, LE, and RS wrote sections of the manuscript. All authors contributed to manuscript revision, proofreading, and approved the submitted version.
