# Supplementary material for: Kynurenic acid as a biochemical factor underlying the association between Western-style diet and depression: A cross-sectional study
Source: Front Nutr. 2022 Oct 10;9:945538. doi: 10.3389/fnut.2022.945538 (PMC9589270; doi:10.3389/fnut.2022.945538)
Supplement: Supplementary file 2 [file Data_Sheet_1.docx]

**Figure Captions:**

**Supplemental Figure 1.** Flow chart of sample selection and missing values. QA, quinolinic acid; KA, kynurenic acid.
