# Supplementary material for: Kynurenic acid as a biochemical factor underlying the association between Western-style diet and depression: A cross-sectional study
Source: Front Nutr. 2022 Oct 10;9:945538. doi: 10.3389/fnut.2022.945538 (PMC9589270; doi:10.3389/fnut.2022.945538)
Supplement: Supplementary file 3 [file Table_1.docx]

**Supplemental Table 1: Summary statistics of immune markers and KP metabolites based on DFS groups.**

|  | **Low DFS**  **(**n=68**)** | **Mid DFS**  **(**n=55**)** | **High DFS**  **(**n=46**)** | ***F*-statistic** | ***p*-value** |
| --- | --- | --- | --- | --- | --- |
| **Immune markers** |  |  |  |  |  |
| C-Reactive Protein (CRP), pg/mL | 24.68 + 54.06 | 25.00 + 38.79 | 30.62 + 73.58 | 0.66 | 0.516 |
| Interleukin (IL)-6, ng/mL | 1.12 + 2.79 | 1.80 + 5.20 | 2.46 + 9.62 | 0.28 | 0.759 |
| **KP metabolites** |  |  |  |  |  |
| Tryptophan (TRP), μmol/L | 5.31 + 2.98 | 5.27 + 4.40 | 6.13 + 3.88 | 1.75 | 0.177 |
| Kynurenine (KYN), μmol/L | 0.31 + 0.14 | 0.29 + 0.20 | 0.36 + 0.26 | 1.86 | 0.160 |
| Kynurenic acid (KA), μmol/L | 1.02 + 0.43 | 0.95 + 0.55 | 0.82 + 0.43* | 3.86 | **0.023** |
| 3-Hydroxykynurenine (3HK), nmol/L | 54.86 + 35.10 | 61.36 + 56.50 | 71.35 + 64.56 | 0.69 | 0.502 |
| 3-Hydroxyanthranilic acid (3HAA), nmol/L | 159.88 + 123.24 | 196.27 + 173.54 | 178.24 + 134.41 | 2.67 | 0.072 |
| Picolinic acid (PA), nmol/L | 63.37 + 30.24 | 81.93 + 119.75 | 81.82 + 76.06 | 2.05 | 0.132 |
| Quinolinic acid (QA), nmol/L | 671.23 + 499.34 | 592.02 + 258.60 | 661.90 + 262.09 | 1.03 | 0.359 |
| **KP Ratio** |  |  |  |  |  |
| IDO/TDO Activity (x10^3^) | 77.68 + 75.67 | 78.67 + 113.91 | 64.16 + 40.65 | 0.72 | 0.487 |
| KAT Activity (x10^-2^) | 40.83 + 27.40 | 43.87 + 31.88 | 31.00 + 20.35*^@^ | 4.10 | **0.018** |
| KMO Activity | 179.26 + 91.42 | 215.47 + 111.89 | 214.53 + 127.30 | 2.12 | 0.123 |
| KYNU Activity (x10) | 37.99 + 37.87 | 44.01 + 38.60 | 35.21 + 24.58 | 1.84 | 0.163 |
| KA/QA ratio (x10) | 18.90 + 11.61 | 17.96 + 10.84 | 13.02 + 7.42*^@^ | 5.70 | **0.004** |

IDO, Indoleamine 2,3-dioxygenase; TDO, Tryptophan dioxygenase; KAT, Kynurenine aminotransferase; KMO, Kynurenine 3-monooxygenase; KYNU, Kynureninase; IDO/TDO activity is defined by KYN/TRP ratio; KYAT activity is defined by KA/KYN ratio; KMO activity is defined by 3HK/KYN; KYNU activity is defined by 3HAA/3HK ratio; DFS, dietary fat and free sugar screener score. All data were presented in mean + SD and corrected to per mmol/L creatinine. All variables were log_2_-transformed prior to applying comparison statistical analyses. * denote *p* < 0.05, using Sidak post-hoc analysis in comparison to low DFS group. @ denote *p* < 0.05 using Sidak post-hoc analysis in comparison to mid DFS. *Indicate p value close to 0.05. Significant *p*-value (<0.05) are denoted in bold.
