# Supplementary material for: Kynurenic acid as a biochemical factor underlying the association between Western-style diet and depression: A cross-sectional study
Source: Front Nutr. 2022 Oct 10;9:945538. doi: 10.3389/fnut.2022.945538 (PMC9589270; doi:10.3389/fnut.2022.945538)
Supplement: Supplementary file 4 [file Table_2.docx]

**Supplemental Table 2: Correlations of depression and biomarkers measures by univariate and covariate analyses.**

|  | **Univariate analysis** | | |  | **Covariate analysis*** | | |
| --- | --- | --- | --- | --- | --- | --- | --- |
|  | **β (95% CI)** | ***R*^2^** | ***p*-value** |  | **Adjusted**  **β (95% CI)** | **Adjusted *R*^2^** | ***p*-value** |
| **Immune marker** |  |  |  |  |  |  |  |
| CRP, pg/ml per mmol Cr | 0.015 (-0.031 – -0.061) | 0.003 | 0.521 |  | 0.014 (-0.032 – 0.059) | 0.122 | 0.554 |
| IL-6, fg/ml per mmol Cr | -0.042 (-0.074 – -0.009) | 0.037 | **0.013** |  | -0.041 (-0.073 – -0.009) | 0.154 | **0.013** |
|  |  |  |  |  |  |  |  |
| **KP metabolites** |  |  |  |  |  |  |  |
| TRP, μmol/L per mmol Cr | 0.038 (-0.115 – 0.191) | 0.001 | 0.625 |  | -0.036 (-0.188 – 0.116) | 0.122 | 0.644 |
| KYN, μmol/L per mmol Cr | -0.029 (-0.194 – 0.135) | 0.001 | 0.724 |  | -0.091 (-0.254 – 0.073) | 0.127 | 0.277 |
| KA, nmol/L per mmol Cr | -0.302 (-0.463 – -0.142) | 0.077 | **<0.0001** |  | -0.266 (-0.430 – -0.103) | 0.174 | **0.002** |
| 3HK, nmol/L per mmol Cr | 0.076 (-0.053 – 0.205) | 0.008 | 0.248 |  | 0.015 (-0.113 – 0.144) | 0.121 | 0.814 |
| 3HAA, nmol/L per mmol Cr | 0.037 (-0.090 – 0.165) | 0.002 | 0.564 |  | -0.004 (-0.131 – 0.123) | 0.120 | 0.952 |
| PA, nmol/L per mmol Cr | -0.076 (-0.269 – 0.117) | 0.004 | 0.439 |  | -0.126 (-0.317 – 0.065) | 0.130 | 0.195 |
| QA, nmol/L per mmol Cr | 0.059 (-0.150 – 0.269) | 0.002 | 0.577 |  | 0.028 (-0.239 – 0.184) | 0.121 | 0.795 |
|  |  |  |  |  |  |  |  |
| **KP Ratio** |  |  |  |  |  |  |  |
| IDO/TDO Activity | -0.062 (-0.213 – 0.089) | 0.004 | 0.419 |  | -0.038 (-0.183 – 0.107) | 0.122 | 0.605 |
| KAT Activity | -0.156 (-0.280 – -0.032) | 0.036 | **0.014** |  | -0.100 (-0.228 – 0.027) | 0.133 | 0.123 |
| KMO Activity | 0.183 (0.004 – 0.361) | 0.024 | **0.045** |  | 0.143 (-0.038 – -0.325) | 0.134 | 0.121 |
| KYNU Activity | -0.031 (-0.149 – 0.087) | 0.002 | 0.607 |  | -0.016 (-0.132 – 0.100) | 0.121 | 0.789 |
| KA/QA ratio | -0.289 (-0.437 – -0.141) | 0.082 | **<0.0001** |  | -0.217 (-0.372 – -0.063) | 0.161 | **0.006** |

CRP, C-Reactive Protein; Cr, Creatinine; IL-6, Interleukin-6; TRP, Tryptophan; KYN, Kynurenine; 3HK, 3-Hydroxykynurenine; 3HAA, 3-Hydroxyanthranilic acid; PA, Picolinic acid; QA, Quinolinic acid; IDO, Indoleamine 2,3-dioxygenase; TDO, Tryptophan dioxygenase; KAT, Kynurenine aminotransferase; KMO, Kynurenine 3-monooxygenase; KYNU, Kynureninase; IDO/TDO activity is defined by KYN/TRP ratio; KAT activity is defined by KA/KYN ratio; KMO activity is defined by 3HK/KYN; KYNU activity is defined by 3HAA/3HK ratio; *General Linear Model (GLM) was performed to adjust for demographic factors including age, sex and DFS and physical activity. β denotes the beta coefficient of the regression model and *R*^2^ refers to the coefficient of determinant indicating the goodness-of-fit of the regression analysis. All variables were log_2_-transformed prior to analysis. Significant *p*-value (<0.05) are denoted in bold.
