# Supplementary material for: Kynurenic acid as a biochemical factor underlying the association between Western-style diet and depression: A cross-sectional study
Source: Front Nutr. 2022 Oct 10;9:945538. doi: 10.3389/fnut.2022.945538 (PMC9589270; doi:10.3389/fnut.2022.945538)
Supplement: Supplementary file 5 [file Table_3.docx]

**Supplemental Table 3: Modelling of association between depression, diet, exercise and the KP.**

|  | **Model 1 (*R*^2^ = 0.2030)** | | | **Model 2 (*R*^2^ = 0.2178)** | | | **Model 3** | | |
| --- | --- | --- | --- | --- | --- | --- | --- | --- | --- |
|  | **β** | **95% CI** | ***p*-value** | **β** | **95% CI** | ***p*-value** | **β** | **95% CI** | ***p*-value** |
| **Demographic factors** |  |  |  |  |  |  |  |  |  |
| Age | -0.257 | -0.834 – 0.321 | 0.381 | -0.250 | -0.824 – 0.324 | 0.391 | -0.250 | -0.804 – 0.304 | 0.377 |
| Sex | 0.052 | -0.212 – 0.316 | 0.699 | 0.012 | -0.255 – 0.278 | 0.931 | 0.012 | -0.245 – 0.269 | 0.929 |
| BMI | -0.022 | -0.069 – 0.026 | 0.376 | -0.022 | -0.070 – 0.025 | 0.356 | -0.022 | -0.068 – 0.024 | 0.341 |
|  |  |  |  |  |  |  |  |  |  |
| **Lifestyle factors** |  |  |  |  |  |  |  |  |  |
| Physical activity | -0.095 | -0.175 – -0.016 | **0.019** | -0.086 | -0.165 – -0.006 | **0.036** | -0.086 | -0.163 – -0.008 | **0.030** |
| DFS |  |  |  |  |  |  |  |  |  |
| as score |  |  |  | 0.684 | 0.303 – 1.066 | **0.001** | 0.6884 | 0.316 – 1.053 | **<0.0001** |
| Mid Group^¤^ | 0.525 | 0.225 – 0.824 | **0.001** | 0.510 | 0.212 – 0.808 | **0.001** | 0.510 | 0.223 – 0.798 | **0.001** |
| High Group^¤^ | 0.556 | 0.238 – 0.874 | **0.001** | 0.561 | 0.245 – 0.877 | **0.001** | 0.561 | 0.256 – 0.866 | **<0.0001** |
|  |  |  |  |  |  |  |  |  |  |
| **Immune marker** |  |  |  |  |  |  |  |  |  |
| IL-6, fg/mL per mmol/L Cr | - | - | - | -0.028 | -0.060 – 0.004 | 0.087 | -0.028 | -0.058 – 0.003 | 0.077 |
|  |  |  |  |  |  |  |  |  |  |
| **KP marker** |  |  |  |  |  |  |  |  |  |
| KA, nmol/L per mmol/L Cr | -0.245 | -0.408 – -0.082 | **0.004** | -0.214 | -0.380 – -0.048 | **0.012** | -0.214 | -0.374 – -0.053 | **0.009** |

BMI, Body Mass Index; DFS, dietary fat and free sugar screener score; IL-6, interleukin-6; KA, kynurenic acid; Cr, creatinine. The β denotes the beta coefficient of the regression model and *R*^2^ refers to the coefficient of determinant indicating the goodness-of-fit of the regression analysis. All variables except BMI were log_2_-transformed prior to regression analysis. ¤ denote variable was chosen to be analysed primarily as a categorical variable rather than continuous variable. Comparisons were made to Low DFS group as baseline reference group. Only variables with *p*-value <0.05 were considered important in predicting the severity of the depressive symptoms. Models 1 and 2 were analysed using multivariate linear regression modelling while Model 3 was analysed with mix-effects linear regression where the two separate RCTs was the random effect. Specification of the model as per described in statistical analysis section. Significant *p*-value (<0.05) are denoted in bold.
