# Supplementary material for: Kynurenic acid as a biochemical factor underlying the association between Western-style diet and depression: A cross-sectional study
Source: Front Nutr. 2022 Oct 10;9:945538. doi: 10.3389/fnut.2022.945538 (PMC9589270; doi:10.3389/fnut.2022.945538)
Supplement: Supplementary file 6 [file Table_4.docx]

**Supplemental Table 4: Impact of diet on biomarkers measures by logistic regression.**

| **Biomarkers** | **TN vs TP vs FN Vs FP^1^**  **(n=166)** | |  | **TN Vs TP^2^**  **(n=103)** | |
| --- | --- | --- | --- | --- | --- |
|  | **OR (95% CI)** | ***p*-value** |  | **OR (95% CI)** | ***p*-value** |
| Age | 1.03 (0.94 – 1.12) | 0.522 |  | 0.92 (0.78 – 1.10) | 0.369 |
| Sex | 1.12 (0.57 – 2.21) | 0.750 |  | 1.54 (0.57 – 4.19) | 0.399 |
| BMI | 0.96 (0.85 – 1.09) | 0.517 |  | 0.98 (0.81 – 1.17) | 0.805 |
| Physical Activity | 0.81 (0.66 – 1.00) | **0.046** |  | 0.78 (0.58 – 1.06) | 0.110 |
| IL-6 | 1.01 (1.00 – 1.03) | 0.064 |  | 1.03 (1.00 – 1.05) | **0.024** |
| KA | 0.47 (0.29 – 0.76) | **0.002** |  | 0.34 (0.210 – 0.983) | **0.002** |

BMI, Body Mass Index; IL-6, Interleukin-6; KA, Kynurenic acid; OR, Odd ratio; CI, confidence interval. In this stratification analysis, we grouped the cohort into 4 groups: True negative (TN) cases refer to those who have healthier diet and not depressed; True positive (TP) cases refer to those who have unhealthier diet and depressed; False negative (FN) cases refer to those who have unhealthier diet but not depressed; and False positive (FP) cases refer to those have healthy diet but depressed. Diet was based on cut-off from sample mean of DFS score at 58. Those with DFS >58 was considered unhealthy. Depression was defined by DASS-21-D score of > 7. Multivariate logistic regression was used to model the outcomes of TN, TP, FN and FP in model 1 (denote by 1) and only TN and TP in model 2 (denote by 2). Model 1, *R*^2^ = 0.078 and Model 2, *R*^2^ = 0.163. Significant *p*-value (<0.05) are denoted in bold.
